# The Importance of Patient Involvement in Stroke Rehabilitation

**DOI:** 10.1371/journal.pone.0157149

**Published:** 2016-06-10

**Authors:** Hanne Kaae Kristensen, Malin Tistad, Lena von Koch, Charlotte Ytterberg

**Affiliations:** 1 Health Sciences Research Centre, University College Lillebaelt, Odense, Denmark; 2 Research Unit in Rehabilitation, Institute of Clinical Research, University of Southern Denmark, Odense, Denmark; 3 Department of Neurobiology, Care Sciences and Society, Karolinska Institutet, Huddinge, Sweden; 4 School of Education, Health and Social Studies, Dalarna University, Falun, Sweden; 5 Department of Neurology, Karolinska University Hospital, Stockholm, Sweden; University of Glasgow, UNITED KINGDOM

## Abstract

**Objective:**

To investigate the perceived needs for health services by persons with stroke within the first year after rehabilitation, and associations between perceived impact of stroke, involvement in decisions regarding care/treatment, and having health services needs met.

**Method:**

Data was collected, through a mail survey, from patients with stroke who were admitted to a university hospital in 2012 and had received rehabilitation after discharge from the stroke unit. The rehabilitation lasted an average of 2 to 4.6 months. The Stroke Survivor Needs Survey Questionnaire was used to assess the participants' perceptions of involvement in decisions on care or treatment and needs for health services in 11 problem areas: mobility, falls, incontinence, pain, fatigue, emotion, concentration, memory, speaking, reading, and sight. The perceived impact of stroke in eight areas was assessed using the Stroke Impact Scale (SIS) 3.0. Eleven logistic regression models were created to explore associations between having health services needs met in each problem area respectively (dependent variable) and the independent variables. In all models the independent variables were: age, sex, SIS domain corresponding to the dependent variable, or stroke severity in cases when no corresponding SIS domain was identified, and involvement in decisions on care and treatment.

**Results:**

The 63 participants who returned the questionnaires had a mean age of 72 years, 33 were male and 30 were female. Eighty percent had suffered a mild stroke. The number of participants who reported problems varied between 51 (80%, mobility) and 24 (38%, sight). Involvement in decisions on care and treatment was found to be associated with having health services needs met in six problem areas: falls, fatigue, emotion, memory, speaking, and reading.

**Conclusions:**

The results highlight the importance of involving patients in making decisions on stroke rehabilitation, as it appears to be associated with meeting their health services needs.

## Introduction

Stroke is the most common cause of morbidity and long-term disability in Europe [1]. It often imposes a considerable change on people’s lives and is an economic burden to society [1]. Stroke survivors often experience physical, cognitive, social, and emotional consequences after stroke [1–3]. The healthcare offered after stroke aims to ease and support restoration of functioning and/or adaptation to disability, and to enable people with stroke to achieve optimal social integration [3–5]. Therefore, rehabilitation is an important part of these services. However, stroke survivors find themselves left with substantial activity and participation limitations and/or in need of daily help. They report long-term unmet needs for rehabilitation of up to 8 years after a stroke [6–10].

To develop high-quality healthcare for stroke survivors it is essential to have a common understanding of the needs, experiences, and priorities of those living with the results of stroke [2, 11,12]. Reviews of self-reported problems experienced by stroke survivors and their carers place strong emphasis on the social aspects of re-establishing former identities and resuming previous occupational, family, social and recreational roles [4]. Many stroke survivors and carers experience social isolation and worsening relationships with their spouses and family [13]. In addition, emotional problems, typically long-standing depressions and anxiety are common [13]. Even so, rehabilitation services seem to be aimed mostly at regaining function and, less, at enabling social participation and regaining former roles and responsibilities [11,14–17]. A survey on the prevalence of unmet needs of community-dwelling stroke survivors across the United Kingdom 1–5 years after the stroke was conducted. The survey showed that nearly half of the survivors reported one or more unmet needs related to problems with mobility, pain, fatigue, memory, and concentration [9]. Other studies have found that stroke survivors also report unmet needs related to activities of daily living (ADL). These needs are: social participation, mobility aids, home adaptation, housing, accessing financial support and benefits, information, rehabilitation and transport between 1 and 11 years after stroke [2, 7].

Clinical practice based on the best available evidence is recommended to provide high-quality service at all levels of the Danish rehabilitation organisation [4]. Evidence recommends that rehabilitation should be designed as a goal-directed, multidimensional, interdisciplinary, and cooperative practice [3, 4, 18]. International rehabilitation literature recognises a growing appreciation of including patients’ experiences and perspectives in rehabilitation practice. A person-centred practice stresses patients’ engagement, the interpersonal relationship between patient and health professionals, and ethical values [19–21]. Studies have shown that increased engagement and patient participation lead to greater satisfaction for both patient and provider. It also leads to increased adherence to health professionals’ recommendations and improved functioning [19, 22]. This is consistent with a patient, person, or client- centred perspective, which is defined as a joint practice, aimed at enabling cooperation between patients and health professionals. A person-centred perspective entails showing respect, involving and empowering the patients in shared decision-making, acting with and for them to meet their needs, and recognising patients’ experiences and knowledge [19]. Thus, hospital and city policies in Denmark are now stressing implementing structures and health policies to increase the extent to which the rehabilitation services are based on shared decision-making and partnerships between patients and health professionals [4, 23].

Stroke rehabilitation that is based on the stroke survivors’ needs, experiences, and priorities requires extensive knowledge and skills to capture and integrate the stroke survivors' perspectives. Few studies have explored the relationship between patients’ engagement in decision-making on rehabilitation, their perceived functioning, and needs of healthcare [2].

The purpose of this study was to explore the perceived needs for health services by people with stroke within the first year after rehabilitation. Moreover, examination of associations between perceived impact of stroke, involvement in decisions on care/treatment, and having health services needs met were undertaken.

## Materials and Methods

**Fig 1:**
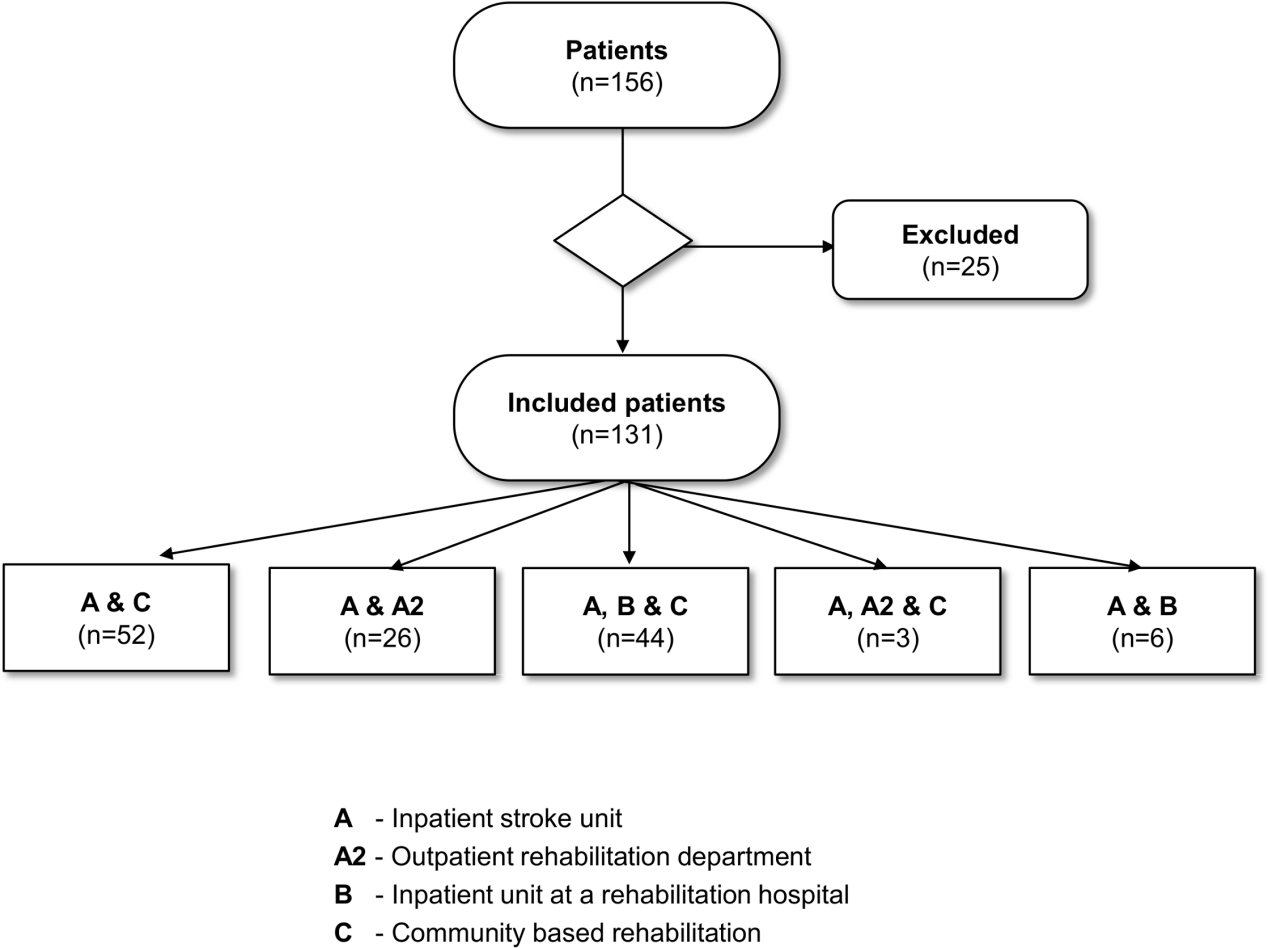
The stroke patients’ rehabilitation pathways.

The study used a survey to investigate patients’ rehabilitation experiences within Danish stroke rehabilitation practise. Data generation was based on the interdisciplinary rehabilitation offered in the different pathways, which comprise the general Danish healthcare service for adult stroke patients in hospitals as well as in community-based settings. Setting A was an in-patient stroke unit located in an acute ward at a university hospital; Setting A2 was an outpatient neurological rehabilitation department located in the same hospital (Setting A); Setting B was an in-patient rehabilitation hospital, exclusively for patients with neurological disorders; and Setting C was a local community-based rehabilitation setting. Stroke rehabilitation was organised so the individual patient could follow one of five different pathways: *pathway 1* included settings A and A2; *pathway 2* settings A, A2 and C; *pathway 3* settings A and B; *pathway 4* settings A, B and C; and *pathway 5* settings A and C. Regardless of the combination of settings, the length of rehabilitation within the pathways consisting of three settings lasted approximately 4 to 4.6 months. The rehabilitation within the pathways that consisted of two settings lasted approximately 2 months. This characteristic was seen regardless of the combinations of settings, see Fig 1.

### Participants

Inclusion criteria were adults aged 18 or over with a stroke diagnosis who had participated in a rehabilitation programme after being discharged from the acute stroke unit at a university hospital in Denmark between May 2012 and December 2012.

All participants underwent interdisciplinary rehabilitation in each of the units included in the rehabilitation pathways. Most participants were discharged from the acute stroke unit within a week and received further rehabilitation in an in-patient rehabilitation hospital exclusively for patients with neurological disorders; and the local community-based rehabilitation setting.

The therapists who offered the rehabilitation were all familiar with Danish national evidence-based guidelines, which they, according to the Danish Board of Health [4], were expected to use in their daily practice.

### Procedure

The occupational therapists and physiotherapists in the stroke unit consecutively considered patients for inclusion in the study and informed the first author of potential participants. The first author then extracted data from the medical records of the units in the rehabilitation pathways. This was done to identify patients who had participated in rehabilitation after being discharged from the stroke unit. The survey instruments were pilot tested to strengthen the validity of the included questionnaires in a Danish context. Stressing variation concerning age, sex, length of rehabilitation and rehabilitation pathway, 13 of the included 131 participants were chosen. The 13 participants were contacted by phone and consented to individual interviews in their own homes after end of rehabilitation by the first author using two questionnaires with closed questions. During the interviews, the participants were asked to consider the aim of the survey and to evaluate the applicability of the questionnaires. All 13 participants considered the questionnaires relevant, satisfactory and understandable in relation to the stroke rehabilitation they had undertaken. The two self-administered questionnaires were then delivered by mail to the remaining 118 participants. The mail included an information and consent letter, and a stamped, addressed envelope for returning the data. Voluntary participation was stressed. Confidentiality in the study was guaranteed and the participants were told they could withdraw at any stage. No reminders were sent. In accordance with Danish legislation on research ethics the research question, design and methods of the study did not require approval by the Research Ethics Committee. The Danish Data Protection Agency, j. no. 2007-41-0836 and the Danish Health and Medicines Authority approved the study. The study followed the directions of the Danish Board of Health.

### Data collection

Data was collected on perceived impact of stroke, perceived involvement in decisions on care/treatment, and perceived health service needs between May 2012 and August 2012. The survey was undertaken between one to 12 months after the participants’ rehabilitation had ended.

Data on sex, age, hemisphere lesion, and stroke severity assessed with the Scandinavian Stroke Scale (SSS) [24, 25] on admission to the stroke unit was extracted from the medical records at the stroke unit retrospectively by the first author. The score range of the SSS is 0–58; scores of 0–25 represent severe, 26–42 moderate and 43–58 mild stroke.

The self-perceived impact of stroke was assessed using the SIS S3.0 [26]. The instrument assesses perceived impact of stroke in eight areas: strength, hand function, ADL, mobility, communication, emotion, memory and thinking, and participation. The SIS comprises 59 items scored from 1 to 5. An algorithm is used to create total scores of 0–100 for each area where 0 represents maximum impact and 100 no impact. SIS also includes a separate question about perceived recovery assessed by a scale from 0–100 where 0 stands for no recovery and 100 is fully recovered.

To assess the participants' perceptions of involvement in decisions on care and treatment, and healthcare needs after stroke, the Stroke Survivor Needs Survey Questionnaire (SSNSQ) was used [9]. The SSNSQ consists of 44 closed questions with response categories to assess level of change or needs for healthcare in the following domains: information about stroke; health after stroke; everyday living; work and leisure; family, friends and support groups; finances and demographic information. In addition, there is one question about involvement in decisions about care and treatment. In the present study, the question about involvement in decisions regarding care and treatment, as well as 11 questions about needs for healthcare were used. The questions about needs dealt with met/unmet needs regarding 11 problems areas: mobility, falls, incontinence, pain, fatigue, emotion, concentration, memory, speaking, reading, and sight. For participants noting a problem in a specific problem area, three response choices were offered: need met, need met to some extent, need unmet. For participants wanting to be involved in decisions about care and treatment, three response choices were offered: involved, involved to some extent, not involved. The participants were asked to consider all rehabilitation related to their stroke when completing the SSNSQ.

### Statistical Analysis

In all analyses the three response choices in the SSNSQ were collected into *need met* versus *need met to some extent* or *need unmet*, and *involved* versus *involved to some extent* or *not involved*. To analyse differences between participants with met and unmet needs concerning the 11 problem areas, the Mann-Whitney U-test was used for numerical data and the Chi-squared test for categorical data. The level of significance was set at p≤0.05.

Eleven logistic regression models were created to explore associations between having health services needs met with regard to each problem area respectively (dependent variable) and the independent variables. In all models the independent variables were: age, sex, SIS domain corresponding to the dependent variable, or stroke severity in cases where no corresponding SIS domain was identified, and involvement in decisions on care and treatment. Both stepwise forward and stepwise backward selections were used where variables with p≤0.05 were entered and those with p≥0.10 were removed. The Statistical Analyses Systems (SAS)® System 9.3, SAS Institute Inc., Cary, NC, USA was used for the statistical analysis.

## Results

In total, 156 consecutive patients were considered for inclusion in the study of which 25 patients were excluded: 5 were deceased, two declined participation and 18 were not referred for further rehabilitation from the acute stroke unit. Questionnaires were sent to the remaining 131 stroke survivors; 70 men and 61 women, aged 25–99, with a mean age of 72. Sixty-three participants answered and returned the questionnaires. The mean age of those who answered the questionnaires was 72 years with a range from 25 to 96 years. Thirty-three of these were men and 30 were women. Of the study participants, 80% had suffered a mild stroke, median SSS score 52. Thirty-one had right sided hemisphere lesions, 25 had a left sided, and seven had lesions in both hemispheres. The mean age of those not returning the questionnaires was also 72 years (range 43–99 years). They were equally divided concerning sex and hemisphere lesions and in the group of stroke survivors who did not return the questionnaires, 55% had suffered a mild stroke.

Table 1 shows the characteristics of participants with met and unmet needs concerning the 11 problem areas categorized with respect to the independent variables, and p values from the univariate analyses. In all problem areas except pain, most of those experiencing a problem reported unmet needs. Participants who felt they had been involved in the decisions regarding their care and treatment were more likely to report having health services needs met concerning seven problem areas: incontinence, pain, fatigue, emotion, concentration, memory, and speaking.

**Table 1:** Characteristics of the participants.

Problem area	1–12 months after stroke		
	Need met	Need met to some extent or Unmet	P value
**Mobility, n (%)**	19 (37)	32 (63)	
Age in years, mean (sd)	19 (37)	73 (9)	0.894
Sex, men/women n	0/8	14/17	0.483
SIS Mobility, mean (sd)	76 (29)	67 (25)	0.118
*Involvement in care and treatment*, *n*			
Involved/Involved to some extent or Not involved	7 /5	7/16	0.110
**Falls, n (%)**	14 (36)	25 (64)	
Age in years, mean (sd)	71 (11)	74 (9)	0.519
Sex, men/women, n	5/8	1/13	0.666
SIS Mobility, mean (sd)	76 (25)	61 (22)	**0.046**
*Involvement in care and treatment*, *n*			
Involved/Involved to some extent or Not involved	6/3	6/13	0.080
**Incontinence, n (%)**	10 (33)	20 (67)	
Age in years, mean (sd)	76 (11)	71 (11)	0.183
Sex, men/women n	3/6	10/10	0.404
Stroke severity, mean (sd)	49 (7)	49 (10)	0.689
*Involvement in care and treatment*, *n*			
Involved/Involved to some extent or Not involved	5/2	2/12	**0.009**
**Pain, n (%)**	19 (51)	18 (49)	
Age in years, mean (sd)	73 (16)	74 (9)	0.684
Sex, men/women, n	9/8	9/9	0.862
Stroke severity, mean (sd)	46 (8)	49 (11)	0.121
*Involvement in care and treatment*, *n*			
Involved/Involved to some extent or Not involved	9/7	2/11	**0.024**
**Fatigue, n (%)**	12 (24)	37 (76)	
Age in years, mean (sd)	71 (9)	70 (12)	.701
Sex, men/women, n	3/8	17/19	0.241
Stroke severity, mean (sd)	51 (8)	48 (10)	0.695
*Involvement in care and treatment*, *n*			
Involved/Involved to some extent or Not involved	9/12	5/18	**<0.001**
**Emotion, n (%)**	9 (24)	28 (76)	
Age in years, mean (sd)	75 (6)	69 (13)	0.132
Sex, men/women, n	4/4	13/14	0.927
SIS Emotion, mean (sd)	79 (19)	70 (24)	0.363
*Involvement in care and treatment*, *n*			
Involved/Involved to some extent or Not involved	6/2	2/18	**0.002**
**Concentration, n (%)**	7 (19)	30 (81)	
Age in years, mean (sd)	75 (11)	69 (12)	0.191
Sex, men/women, n	2/5	15/14	0.271
SIS Memory and thinking, mean (sd)	93 (9)	75 (21)	**0.023**
*Involvement in care and treatment*, *n*			
Involved/Involved to some extent or Not involved	5/2	4/13	**0.028**
**Memory, n (%)**	7 (19)	30 (81)	
Age in years, mean (sd)	71 (10)	72 (13)	0.864
Sex, men/women, n	3/4	16/14	0.618
SIS Memory and thinking, mean (sd)	93 (10)	74 (21)	**0.016**
*Involvement in care and treatment*, *n*			
Involved/Involved to some extent or Not involved	5/2	3/16	**0.006**
**Speaking, n (%)**	8 (31)	18 (69)	
Age in years, mean (sd)	75 (8)	68 (14)	0.160
Sex, men/women, n	2/6	10/12	0.149
SIS Communication, mean (sd)	92 (10)	82 (22)	0.429
*Involvement in care and treatment*, *n*			
Involved/Involved to some extent or Not involved	6/2	3/8	**0.040**
**Reading, n (%)**	7 (22)	24 (78)	
Age in years, mean (sd)	71 (11)	71 (13)	0.982
Sex, men/women, n	2/5	12/12	0.316
Stroke severity, mean (sd)	50 (8)	47 (10)	0.406
*Involvement in care and treatment*, *n*			
Involved/Involved to some extent or Not involved	5/2	5/11	0.074
**Sight, n (%)**	6 (25)	18 (75)	
Age in years, mean (sd)	69 (9)	72 (11)	0.626
Sex, men/women, n	3/3	11/7	0.633
Stroke severity, mean (sd)	47 (10)	51 (6)	0.574
*Involvement in care and treatment*, *n*			
Involved/Involved to some extent or Not involved	4/2	3/9	0.087

Results from the logistic regression analyses are shown in Table 2. The stepwise forward and the stepwise backward selection resulted in the same final models. Involvement in decisions regarding care and treatment was found to be associated with having health services needs met concerning six problem areas: falls, fatigue, emotion, memory, speaking, and reading.

**Table 2:** Final logistic regression models for the association of the independent variables and met needs with regard to the 11 problem areas, odds ratios (OR) and 95% confidence intervals (CI).

*Problem area*		Odds for met needs
Independent variables	Variable categorization	OR (95% CI)
***Mobility***		
Involvement in care and treatment	Involved	3.20 (0.75–13.66)
	Involved to some extent/Not involved	1
*Area under the receiver operating characteristic curve = 0*.*640*		
***Falls***		
SIS mobility	Decreased impact	1.06 (1.00–1.12)
Involvement in care and treatment	Involved	13.40 (1.31–137.53)
	Involved to some extent/Not involved	1
*Area under the receiver operating characteristic curve = 0*.*813*		
***Incontinence***		
Involvement in care and treatment	Involved	10.50 (0.67–165.11)
	Involved to some extent/Not involved	1
*Area under the receiver operating characteristic curve = 0*.*738*		
***Pain***		
Involvement in care and treatment	Involved	9.00 (0.81–100.11)
	Involved to some extent/Not involved	1
*Area under the receiver operating characteristic curve = 0*.*700*		
***Fatigue***		
Involvement in care and treatment	Involved	14.00 (1.84–106.47)
	Involved to some extent/Not involved	1
*Area under the receiver operating characteristic curve = 0*.*787*		
***Emotion***		
Involvement in care and treatment	Involved	22.50 (2.55–198.38)
	Involved to some extent/Not involved	1
*Area under the receiver operating characteristic curve = 0*.*816*		
***Concentration***		
SIS memory and thinking	Decreased impact	1.12 (1.00–1.25)
*Area under the receiver operating characteristic curve = 0*.*832*		
***Memory***		
Involvement in care and treatment	Involved	13.33 (1.71–103.75)
	Involved to some extent/Not involved	1
*Area under the receiver operating characteristic curve = 0*.*778*		
***Speaking***		
Involvement in care and treatment	Involved	8.00 (1.00–63.96)
	Involved to some extent/Not involved	1
*Area under the receiver operating characteristic curve = 0*.*739*		
***Reading***		
Involvement in care and treatment	Involved	20.00 (1.39–287.60)
	Involved to some extent/Not involved	1
*Area under the receiver operating characteristic curve = 0*.*817*		
***Sight***		
Involvement in care and treatment	Involved	3.50 (0.28–43.16)
	Involved to some extent/Not involved	1
*Area under the receiver operating characteristic curve = 0*.*639*		

## Discussion

Stroke is a common, serious, and disabling health problem, and rehabilitation is a major part of patient care [3]. Even after having received rehabilitation there are indications that some stroke survivors continue perceiving unmet needs for healthcare [7]. To our knowledge, this is the first study that has explored and identified an association between stroke survivors’ involvement in decisions on care and treatment and having health services needs met concerning six problem areas: falls, fatigue, emotion, memory, speaking, and reading.

In all problem areas except pain, a majority of those experiencing problems reported unmet needs. This is in line with the results of a previous survey on the prevalence of unmet needs in community-dwelling stroke survivors 1–5 years after stroke [9]. Our results show that unmet needs occur during the first year after stroke. One plausible explanation may be that there is a lack of concurrence between the needs perceived by people with stroke and those identified by health professionals [27–30]. Some unmet needs in the present study might not have been identified by health professionals and targeted for intervention. It is also possible that participants had become aware of needs after the rehabilitation had ended.

The findings from the logistic regression models indicating high odds for having health services needs met in problem areas related to falls, fatigue, emotion, memory, speaking, and reading when the person had been involved in decisions about care and treatment, might indicate that patients’ involvement could be an important contributing factor for a favourable outcome after stroke. In the present study, it is not known whether strategies for involving patients in, for example, shared decision-making or common goal setting, were applied [3]. Shared decision-making has been described as a core ingredient in patient-centred care and a reconciliation between respect for a patient’s autonomy and the power of healthcare professionals [31]. Goal setting is also used to support patients’ autonomy and to improve patient motivation, adherence and improve satisfaction with rehabilitation [32]. In line with this, stroke survivors’ involvement in different aspects of decision-making such as goal setting and translation of goals into therapy plans has previously been reported as empowering and highly appreciated whereas authoritarian attitudes and decision-making processes had a negative influence [33–35]. However, people with stroke are not taking part in decisions about their care to the extent expected [22, 36–38] and the extent to which patients are involved in decision-making is in the hands of professionals as they lead the goal setting processes [39–42]. Nevertheless, an increased involvement by the person with stroke in decision-making and goal setting was achieved after training therapists in engaging patients in shared decision-making [43]. Such training might be needed to achieve a more shared goal setting process. Despite the lack of knowledge about specific methods used in the present study, it seems possible to involve patients in decisions about their care and rehabilitation in ordinary clinical practice which might influence to what extent health service needs are perceived to be met.

No associations were found between involvement in decisions on care and treatment and having health services needs met in problem areas related to mobility, incontinence, pain, concentration, and sight. A systematic review of qualitative studies [33] stressed that physical activity in particular is valued by stroke survivors. Walking and mobility in particular were seen as important forms of physical rehabilitation [33]. Walking and mobility have also been shown to be predictors of returning to pre-stroke levels of participation [44, 45]. Though the majority of the participants in the current study had suffered a mild stroke, they might not have resumed full physical recovery. Consequently, they might have experienced lack of independence and control over their daily lives regardless of whether they had been involved in the decisions on their care and treatment. The results on incontinence and sight problems might indicate that these functions had not been assessed and identified as problem areas, or might have been present already pre-stroke, and therefore not emphasised in the stroke rehabilitation. Conversely, having health services needs met regarding reading problems, an activity that might be related to sight, was associated with involvement in decisions on care and treatment. A believable explanation for these results could be that reading problems might be more easily detected and communicated by the person with stroke. The lack of association between involvement in decisions regarding care and treatment and having health services needs met concerning pain may be explained by the fact that pain after stroke can be difficult to treat satisfactorily and has a significant negative effect on health-related quality of life [46].

A strength of the present study is the self-reported data, which gives a voice to those who are concerned. However, the results should be interpreted with caution bearing in mind that the return rate was 48% and that a larger proportion of those not returning the questionnaires had a moderate-severe stroke. As several studies have reported that people with severe impairments or disability after stroke to a larger extent report unmet needs for e.g. mobility and self-care [8,47], adaptations, physiotherapy, social life [8,9,47,48], therapy [10,11,47,48] and assistance with instrumental ADL [10], a higher return rate from people with moderate/severe stroke in the present study might have affected the findings. Contrary to these studies, severity of stroke or the impact of stroke was in the present study only associated with having health services needs met in two of the problem areas, falls and concentration. In the present study, people with aphasia might also be under-represented, as they may have found it difficult to participate in a survey based on questionnaires. Other limitations are the cross-sectional design as no firm conclusions about the direction of the association can be drawn, the limited sample size and the spread in time points to data collection. Future studies would benefit from a larger and more representative sample.

In conclusion, we found an association between stroke survivors’ self-reported involvement in decisions on care and treatment and having health services needs met for problems related to falls, fatigue, emotion, memory, speaking, and reading. As many countries have adapted policies and regulations about patient-centred care, in which involvement in decision about care and rehabilitation is a core ingredient, the issue is highly relevant but more studies are needed to further explore the association between involvement in decision making and experiences of having health services needs met.
